# Machine learning-based improvement of MDS-CBC score brings platelets into the limelight to optimize smear review in the hematology laboratory

**DOI:** 10.1186/s12885-022-10059-8

**Published:** 2022-09-10

**Authors:** Jaja Zhu, Pierre Lemaire, Stéphanie Mathis, Emily Ronez, Sylvain Clauser, Katayoun Jondeau, Pierre Fenaux, Lionel Adès, Valérie Bardet

**Affiliations:** 1 Service d’Hématologie-Immunologie-Transfusion, Hôpitaux Universitaires Paris Ile De France Ouest, APHP. Paris Saclay, Université Versailles Saint Quentin-Université Paris Saclay, Paris, France; 2 Service d’Hématologie biologique, Hôpitaux Universitaires Saint Louis, Lariboisière, Fernand Widal, Université Paris Diderot, Paris, France; 3 Unité fonctionnelle d’Hématologie clinique, Service de Médecine Interne, APHP. Paris Saclay, Université Versailles Saint Quentin, Paris, France; 4 Service d’Hématologie Seniors, Hôpitaux Universitaires Saint Louis, Lariboisière, Fernand Widal, Université Paris Diderot, Paris, France; 5 Groupe Francophone des Myélodysplasies, Hôpital Saint Louis, Paris, France

**Keywords:** Myelodysplastic syndromes, Smear review, Dysplasia score, Ne-WX, MDS-CBC score, Macroplatelets, Immature platelet fraction

## Abstract

**Background:**

Myelodysplastic syndromes (MDS) are clonal hematopoietic diseases of the elderly characterized by chronic cytopenias, ineffective and dysplastic haematopoiesis, recurrent genetic abnormalities and increased risk of progression to acute myeloid leukemia. A challenge of routine laboratory Complete Blood Counts (CBC) is to correctly identify MDS patients while simultaneously avoiding excess smear reviews. To optimize smear review, the latest generations of hematology analyzers provide new cell population data (CPD) parameters with an increased ability to screen MDS, among which the previously described MDS-CBC Score, based on Absolute Neutrophil Count (ANC), structural neutrophil dispersion (Ne-WX) and mean corpuscular volume (MCV). Ne-WX is increased in the presence of hypogranulated/degranulated neutrophils, a hallmark of dysplasia in the context of MDS or chronic myelomonocytic leukemia. Ne-WX and MCV are CPD derived from leukocytes and red blood cells, therefore the MDS-CBC score does not include any platelet-derived CPD. We asked whether this score could be improved by adding the immature platelet fraction (IPF), a CPD used as a surrogate marker of dysplastic thrombopoiesis.

**Methods:**

Here, we studied a cohort of more than 500 individuals with cytopenias, including 168 MDS patients. In a first step, we used Breiman’s random forests algorithm, a machine-learning approach, to identify the most relevant parameters for MDS prediction. We then designed Classification And Regression Trees (CART) to evaluate, using resampling, the effect of model tuning parameters on performance and choose the “optimal” model across these parameters.

**Results:**

Using random forests algorithm, we identified Ne-WX and IPF as the strongest discriminatory predictors, explaining 37 and 33% of diagnoses respectively. To obtain “simplified” trees, which could be easily implemented into laboratory middlewares, we designed CART combining MDS-CBC score and IPF. Optimal results were obtained using a MDS-CBC score threshold equal to 0.23, and an IPF threshold equal to 3%.

**Conclusions:**

We propose an extended MDS-CBC score, including CPD from the three myeloid lineages, to improve MDS diagnosis on routine laboratory CBCs and optimize smear reviews.

**Supplementary Information:**

The online version contains supplementary material available at 10.1186/s12885-022-10059-8.

## Background

Myelodysplastic syndromes (MDS) mainly occur in older adults (median patient age: 70 years). MDS are clonal hematopoietic diseases characterized by chronic cytopenias, ineffective and dysplastic haematopoiesis, recurrent genetic abnormalities and increased risk of progression to acute myeloid leukemia. Among myeloid neoplasms, the diagnosis of MDS is particularly challenging, especially because of the need to distinguish MDS from the numerous other non-neoplastic causes of cytopenia. The updated World Health Organization (WHO) classification of Tumors of Haematopoietic and Lymphoid Tissues [1] still considers the cytopenia thresholds established in the original International Prognostic Scoring System (IPSS) [2] for risk stratification (hemoglobin concentration < 10 g/dL, platelet count < 100 × 10^9^/L and absolute neutrophil count (ANC) < 1.8 × 10^9^/L). However, a MDS diagnosis can be made in patients with a milder degree of anemia or thrombocytopenia [3]. Using the IPSS thresholds, patients with one or more cytopenias account for 23% of complete and differential blood counts (CBC) in our own experience. Considering the low frequency of MDS [4, 5], the International Society for Laboratory Hematology [6] only recommended a blood smear review if hemoglobin level < 7 g/dL or mean corpuscular volume (MCV) > 105 fL or absolute neutrophil count (ANC) < 1 × 10^9^/L or platelet count < 100 × 10^9^/L. Of course, this approach is based on medico-economic considerations and does not allow optimal screening of MDS. To optimize slide review, cell population data parameters (CPD) have been explored for more than 30 years and have demonstrated their added-value in the laboratory diagnostic work-up of MDS [7]. The latest generations of hematology analyzers provide new diagnostic tools with an increased ability to screen MDS from a CBC. A first seminal work from Boutault et al. in 2018 [8] described the “MDS-CBC score” on hematology analyzers from the XN™-series (Sysmex®). This score was established on a cohort of 109 newly diagnosed MDS patients and 399 cytopenic patients older than 50 years. It incorporated three parameters: ANC, structural neutrophil dispersion (Ne-WX) and MCV and was calculated as soon as a cytopenia was detected (hemoglobin < 13 g/dL in men or < 12 g/dL in women, platelet count < 150 × 10^9^/L, ANC < 1.8 × 10^9^/L). Structural neutrophil dispersion is increased in the presence of hypogranulated/degranulated neutrophils, a hallmark of dysplasia in the context of MDS or chronic myelomonocytic leukemia [9]. The combination of these parameters in a multiparametric equation allowed the generation of a “MDS-CBC score” with a sensitivity of 86% and a specificity of 88% for the screening of MDS. More recently, other studies relying on different hematology analyzer technologies confirmed the usefulness of CPD in the assessment of MDS. Two studies were performed on Beckman Coulter analyzers, which measure CBC and differential based on a combination of three physical parameters: flow cell volume (V), conductivity (C) and light scatter measurements (S) obtained by the VCS flow technology. The first study by Shestakova et al. [10] identified neutrophil VCS parameters and especially the degree of heterogeneity of neutrophil upper median angle light scatter (SD-UMALS-NE) as the most predictive of MDS. A second study from Ravalet et al. [11] confirmed the interest of these CPD parameters and proposed an algorithm to calculate the MDS-LS (MDS likelihood score), based on 10 leukocyte parameters. A similar study based on CPD from the Abbott Alinity hq analyzer [12] identified on a large cohort of patients (345 patients including 162 non-MDS cytopenias) leukocyte CPD but also percentage of macrocytic red blood cells (RBCs), hemoglobin distribution width (HDW) and platelet distribution width (PDW) as the most sensitive parameters to differentiate MDS from other forms of cytopenias.

Although this recent study proposed to include PDW, a parameter derived from platelets, we were intrigued by the fact that most of the previous reports only focused on parameters derived from leukocytes or RBCs. Of course, anemia is the most frequent symptom of patients suffering from MDS and neutrophil dysplasia remains the most specific MDS hallmark on the blood smear, but in our opinion, platelets could have been unfairly left out, perhaps due to technological issues. Macroplatelets are a morphological feature of MDS and we wondered if the previously published MDS-CBC score could be improved using the immature platelet fraction (IPF). IPF is a valuable diagnostic parameter reflecting thrombopoiesis, useful for identifying peripheral thrombocytopenia in a blood sample [13]. A high IPF percentage is indicative of platelet turnover arising from increased destruction, consumption or recovery from thrombocytopenia but can also be observed in MDS [14, 15] or inherited macrothrombocytopenia [16].

In this paper, we evaluated on a cohort of more than 500 individuals, including 168 MDS patients, the performance of the MDS-CBC score, relative to the MDS sub-type, the presence and type of cytopenia. In a second step, we used Breiman’s random forests algorithm, a machine-learning approach, to identify the most relevant parameters for MDS prediction and propose an extended MDS-CBC score, including CPD from the three myeloid lineages.

## Patients and methods

### Patients

Demographic, biological and clinical characteristics of the cohort are described in Supplementary Table 1. A total of five hundred and twenty-five patients with one or more cytopenias and a suspicion of MDS were enrolled in this prospective bicentric study from January 2020 to November 2021 at Saint Louis and Ambroise Paré University Hospitals (France). At the end of the diagnostic work-up (including bone marrow examination and cytogenetic analyses for two hundred and sixty-three patients), MDS diagnosis was established in one hundred and sixty-eight patients and excluded in three hundred and fifty-seven patients with non-clonal cytopenias. Characteristics of the MDS cohort (MDS diagnosis according to the 2017 WHO classification criteria [1] and revised International Prognostic Scoring System [3]) are described in Supplementary Table 2. Most frequent causes of non-clonal cytopenias disclosed were vitamin deficiencies, anemia of chronic inflammatory diseases, endocrinological diseases, chronic kidney disease, gastrointestinal diseases or drug-induced anemia.

### Methods

Blood samples from MDS patients and controls were collected in standardized tubes containing potassium ethylenediaminetetraacetate (EDTA) as anticoagulant. CBC analyses were performed using the Sysmex® XN-10™ hematology analyzer (Sysmex® Corporation, Kobe, Japan) equipped with the PLT-F channel to allow measurement of the IPF. MDS-CBC score was calculated as previously published [8].

### Statistics

Categorical and continuous variables are reported as numbers and percentages, or median interquartiles, respectively. Association between MDS diagnosis and categorical and continuous parameters was evaluated using chi-square test and Mann-Whitney test, respectively. All tests were two-sided at a significance level of *p* < 0.05. All analyses were performed using R statistical software V.3.3.2 (R Foundation for Statistical Computing, Vienna, Austria). We further applied machine-learning techniques to visualize and investigate the pattern between MDS diagnosis and several variables. Contribution of each biological variable, including CPD, to the diagnosis of MDS was investigated with random forest models using the randomForestSRC package. The importance of each variable was evaluated with the Variable Importance (VIMP) estimates. The relative contribution of each variable was evaluated by summing the VIMP of all covariates. The CARET (Classification And REgression Training) package was then used to evaluate, using resampling, the effect of model tuning parameters on performance and choose the “optimal” model across these parameters. Random forest models and CART (Classification And Regression Trees) were built and Receiver Operating Characteristic (ROC) curves were used to illustrate the diagnostic ability of classification trees in the machine learning methodology. All statistical analyses were performed with R 4.0.3 (http://www.R-project.org/).

## Results

### Characteristic of the study population

The studied population included 357 patients presenting with non-clonal cytopenias (controls) and 168 patients with MDS. Univariate analysis of baseline characteristics of the cohort revealed eight parameters associated with MDS diagnosis (Supplementary Table 1). MDS patients were slightly older than non-clonal cytopenic patients (78 [71–84] versus 71 years [62–80], *p* < 10^− 4^) (Supplementary Table 1) and more frequently from the male gender. The frequency of anemia was similar between both groups (93.4% versus 93.8%), despite slightly lower hemoglobin levels in MDS patients (9.6 [8.4–11.0] versus 10.5 g/dL [9.5–11.5], *p* = 0.0007) and higher mean corpuscular volume (MCV) (94 [87–102] versus 87 fL [80–93], *p* < 10^− 4^). MDS patients more frequently harbored neutropenia or thrombocytopenia compared to non-clonal cytopenia (45.8% versus 10.1 and 66.7% versus 18.5%, respectively) which was reflected by lower ANC (1.9 [1.0–2.8] versus 5.5 [3.2–8.8], *p* < 10^− 4^) and platelet count (113 [54–177] versus 438 [185–526], *p* < 10^− 4^) (Supplementary Table 1 and Supplementary Fig. 1). As expected, Mean Platelet Volume (MPV) was also significantly increased in MDS patients (11.4 [10.6–12.5] versus 9.8 [9.3–10.4], p < 10^− 4^). Due to analytical interference related to the presence of macroplatelets, MPV was only available in 75% of MDS patients compared to 99% of non-clonal cytopenias, explaining why we focused on the evaluation of IPF.

### Performance of the MDS-CBC score and morphological parameters

**Fig. 1:**
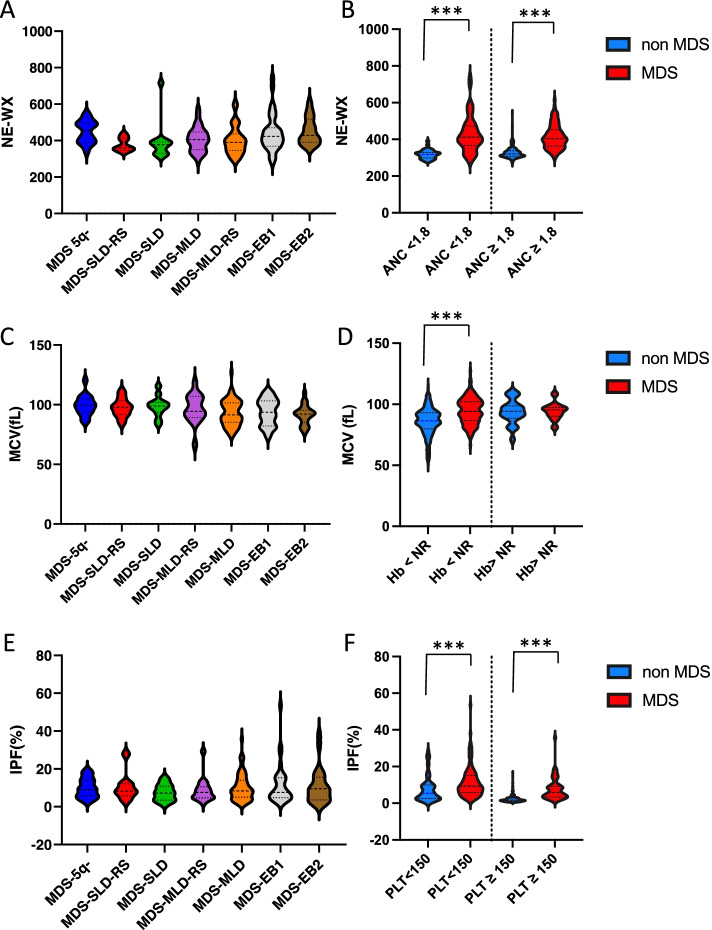
Violin plots of cell population data (CPD) according to MDS subtype (**A**, **C** and **E**) and cytopenia (**B, D** and **F**). **A** Ne-WX varies slightly among MDS subtypes (*p* = 0.005). **B** Ne-WX is significantly increased in MDS patients compared to non-MDS patients, whether they are neutropenic (412 versus 319, *p* < 10^− 4^) or not (404 versus 321, *p* < 10^− 4^). **C** Mean corpuscular volume (MCV) is not significantly different between MDS subtypes (*p* = 0.093). **D** MCV is only increased in MDS versus non-MDS patients with anemia (94 versus 86 fL, p < 10^− 4^). **E** Immature platelet fraction (IPF) is not different between MDS subtypes (*p* = 0.91). **F** IPF is significantly increased in MDS patients compared to non-MDS patients, whether they are thrombopenic (9.3 versus 5.4%, *p* < 10^− 4^) or not (5.8 versus 1.8%, *p* < 10^− 4^)

Using the previously published threshold of 0.2, MDS-CBC score was abnormal in 18.7% of non-clonal cytopenias and 94% of MDS patients (Supplementary Table 1 and Supplementary Fig. 1), thus confirming the high performance of this score, even in such a selected cohort (suspicion of MDS). The performance was globally similar across subtypes of MDS with a slightly decreased diagnostic power in MDS with multilineage dysplasia (MDS-MLD). We then evaluated the diagnostic performance of morphological parameters in the different subtypes of MDS and regarding the presence or absence of cytopenia. Ne-WX was slightly different across MDS subtypes (*p* = 0.005) but not different when comparing high grade MDS versus low grade MDS (Fig. 1A). Interestingly, Ne-WX was constantly increased in MDS patients compared to non-clonal cytopenias whether they were neutropenic or not (Fig. 1B). On the contrary, MCV was not different between MDS subtypes and was only increased in MDS patients showing anemia (Fig. 1C and D). As mentioned earlier, as MPV was not available for all patients, we focused on IPF as a surrogate marker of the presence of dysplastic platelets. IPF was not significantly different between MDS subtypes but, contrary to MCV, was constantly increased in MDS patients whether they harbored thrombocytopenia or not (Fig. 1E and F).

### Machine learning identification of the most contributive parameters for MDS diagnosis

**Fig. 2:**
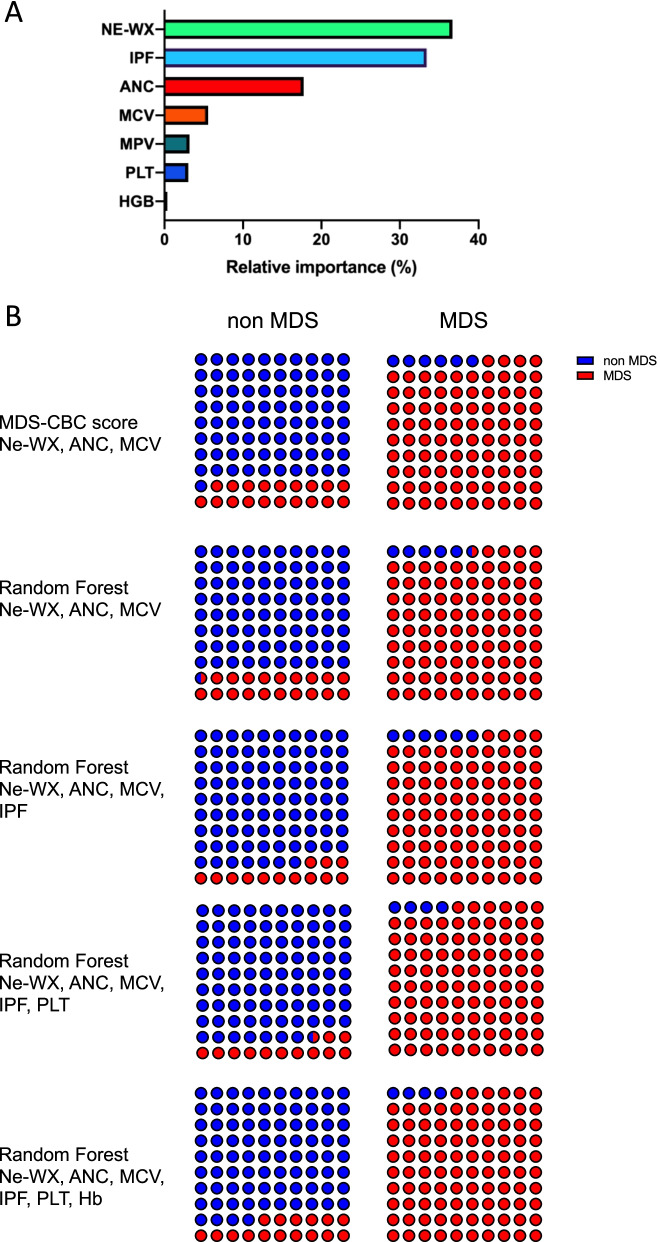
Machine learning-based improvement of the MDS-CBC score. **A** Results of the random forest classification for MDS diagnosis of several CBC parameters, the variable importance (VIMP) parameter is plotted. **B** Performance of the different scores on the cohort to classify patients into 2 groups. Using the MDS-CBC score, 290 non-MDS (81.8%) and 158 MDS (94%) patients are correctly classified. With the 3-parameter RF model, 287 non-MDS (80.4%) and 159 MDS (94.6%) are correctly classified. With the 4-parameter RF model, 310 non-MDS (86.8%) and 158 MDS (94%) are correctly classified. With the 5-parameter RF model, 312 non-MDS (87.4%) and 161 MDS (95.8%) are correctly classified. With the 6-parameter RF model, 300 non-MDS (84%) and 161 MDS (95.8%) are correctly classified

Using Breiman’s random forests (RF) classification, we identified Ne-WX and IPF as the two most discriminatory predictors for MDS diagnosis (Fig. 2A), explaining 37 and 33% of diagnoses respectively. Comparatively, ANC and MCV only contributed to 18 and 6% of diagnoses. We then used the CARET (Classification And REgression Training) package to evaluate the effect of model tuning parameters on performance and choose the “optimal” model across these parameters. A bootstrapping approach was used, splitting the cohort in five sub-cohorts, to cross-validate these models. We started with the three parameters from the MDS-CBC score and added the other parameters one by one to random forest (RF) models, considering parameters with the highest VIMP first. As expected, RF using three parameters (Ne-WX, ANC and MCV) showed similar performances to the MDS-CBC score (Fig. 2B, RF three parameters), with a sensitivity (Se) equal to 95% (94% for the MDS-CBC score) and a specificity (Sp) equal to 80% (81% for the MDS-CBC score). Adding IPF to the model dramatically increased Sp from 80 to 87% while maintaining high Se at 94% (Fig. 2B, RF four parameters). Adding the platelet count to this model slightly increased Se to 96% with a Sp still equal to 87% (Fig. 2B, RF five parameters). Unexpectedly, supplementing the model with hemoglobin level decreased performances with a Sp equal to 84% (Fig. 2B, RF six parameters).

### From artificial intelligence to routine practice: a two-step approach

**Fig. 3:**
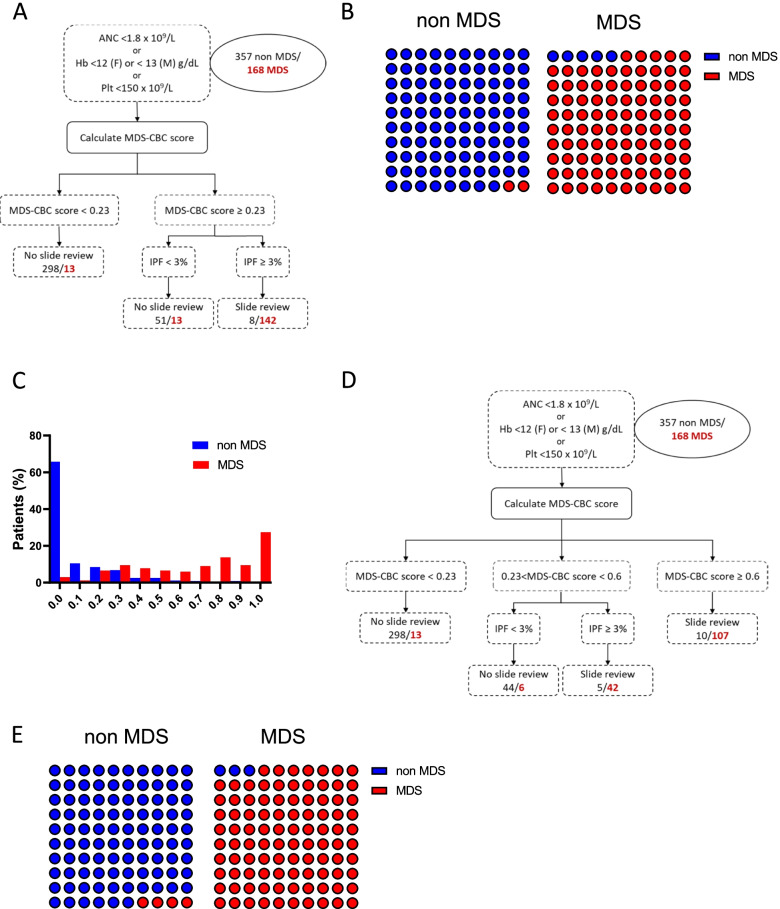
Optimization of the MDS diagnostic work-up. **A** Decision tree based on a threshold equal to 0.23 to guide PLT-F reflex test and an additional cut-off at 3% for IPF to guide slide review. According to this decision tree, slide review was not indicated in 375 patients (including 349 non-MDS and 26 MDS patients) and suggested in 150 patients (including 8 non-MDS and 142 MDS patients). **B** Performance of this decision tree in real-life considering all slide review criteria. 18 of 26 misclassified patients with MDS had additional criteria for slide review. Combining the decision tree and the other criteria for slide review, 349 of non-MDS (97.8%) and 160 of MDS patients (95.2%) were correctly classified. **C** Histogram showing the frequency of MDS and non-MDS patients according to the MDS-CBC score. **D** Decision tree based on a threshold equal to 0.23 and inferior to 0.6 to guide PLT-F reflex test and an additional cut-off at 3% for IPF to guide slide review. Slide review was not indicated in 361 patients (including 342 non-MDS and 19 MDS patients) and suggested in 164 patients (including 15 non-MDS and 149 MDS patients). **E** Performance of this decision tree in real-life considering all slide review criteria. 13 of 19 misclassified patients with MDS had additional criteria for slide review. Combining the decision tree and the other criteria for slide review, 342 of non-MDS (95.8%) and 162 of MDS patients (96.4%) were correctly classified

We then used the CARET package to design CART (Classification And Regression Trees). To obtain “simplified” trees, which could be easily used with the laboratory middleware, we only introduced MDS-CBC score and IPF into the model. Two CART were proposed with similar performances, one with three levels of decision (data not shown) and one with a two-step algorithm (Fig. 3A). Strikingly, the MDS-CBC score threshold proposed by these two algorithms for classification was 0.23, very close to the published threshold of 0.2 by Boutault et al. In the two-step algorithm, if MDS-CBC score was inferior or equal to 0.23, no additional testing or slide review was requested (311 patients including thirteen MDS patients). When MDS-CBC score was superior to 0.23, an IPF threshold equal to 3% was proposed by the machine-learning model to stratify patients (Fig. 3A). 64 patients had an IPF inferior to 3 including only thirteen MDS patients, whereas 142 of 150 patients with an IPF superior or equal to 3 were MDS patients (Fig. 3A). Se of this model was 84.5% (95%CI: 78.3–89.2) and Sp 97.8 (95%CI: 95.6–98.9), whereas positive predictive value (PPV) and negative predictive value (NPV) were equal to 94.7 (95%CI: 89.8–97.3) and 93.1 (95%CI: 90–95.2), respectively. Among the thirteen MDS patients with a score < 0.23, 10 had other criteria for slide review (analyzer flag or ANC < 1.5 × 10^9^/L or Hb < 80 g/L or platelets < 100 × 10^9^/L) as well as eight of the thirteen patients with a score ≥ 0.23 and IPF < 3%. At the end of this diagnostic work-up, 349 of non-MDS (97.8%) and 160 of MDS patients (95.2%) were correctly classified (Fig. 3B). Since machine-learning models do not integrate medico-economic considerations, we wondered if we could determine a threshold beyond which IPF would have no significant benefit, thus allowing saving on PLT-F reagent. Plotting histogram frequency of diagnosis (Fig. 3C) showed that in patients with a MDS-CBC score equal or superior to 0.6 (22% of the cohort), most patients were MDS patients (91%). Considering that the sensitivity of MDS-CBC score was excellent above the 0.6 threshold, we therefore proposed an alternative two-step algorithm illustrated in Fig. 3D. If the MDS-CBC score was inferior to 0.23, there was no indication for slide review for MDS suspicion. A score between 0.23 and 0.6 triggered PLT-F on the analyzer for measurement of IPF, then if IPF was below 3% there was no indication for slide review, if IPF was equal to or superior to 3%, a slide review was required for suspicion of MDS. As previously mentioned, MDS-CBC scores superior or equal to 0.6 were highly predictive of MDS and needed a slide review. This new algorithm, the “extended MDS-CBC (e-MDS-CBC) score”, had high sensitivity: 88.7 (95%CI: 83–92.6) and specificity: 95.8 (95%CI: 93–97.4) with a PPV of 90.8 (95%CI: 85.5–94.4) and a NPV of 94.7 (95%CI: 91.9–96.6) with a reasonable cost. Six MDS patients had a score between 0.23 and 0.6 and IPF inferior to 3, none had a flag on the analyzer but three had other slide review criteria. At the end of this diagnostic work-up, 342 of non-MDS (95.8%) and 162 of MDS patients (96.4%) were correctly classified (Fig. 3E).

## Discussion

In this paper, we evaluated on a cohort of 168 MDS patients and 357 non-clonal cytopenias, the performance of the MDS-CBC score, relative to the MDS sub-type, the presence and type of cytopenia. On this large cohort, we confirmed the high diagnostic performance of this score regardless of MDS subtype. By looking more closely at the parameters of this score, we observed that Ne-WX, a hallmark of dysplasia, was constantly increased in MDS patients whether they harbored neutropenia (45.8% of patients) or not. On the contrary, MCV was only increased in MDS patients showing anemia. As discussed earlier, our aim was to evaluate if IPF, a morphological parameter derived from platelets, could be a hallmark of the presence of macroplatelets and more generally, a feature of dysplastic thrombopoiesis in MDS patients. IPF has been widely studied as a diagnostic tool in a variety of settings (immune thrombocytopenia, thrombotic thrombocytopenic purpura, coronary syndromes, sepsis, liver disease) [17], however literature is scarce regarding its use in the diagnosis of MDS [14, 15]. Interestingly, in our study, IPF was significantly increased in MDS patients compared to non-clonal cytopenias regardless of MDS subtype (Fig. 1D) and whether thrombocytopenia was present or not (Fig. 1E).

We then used Breiman’s random forests algorithm, a machine-learning approach, to identify the most relevant parameters for MDS prediction among routine and research parameters from CBC. In connection with our observation that Ne-WX was consistently increased in MDS regardless of neutrophil count, Ne-WX was the best contributor for MDS diagnosis explaining 37% of diagnoses (Fig. 2A). Conversely, MCV only accounted for 6% of diagnoses despite anemia being the most frequent cytopenia in MDS (93.4% in our cohort). This probably reflects the lack of specificity of macrocytosis in MDS considering reactive conditions of anemia where macrocytosis is frequent, such as endocrinological diseases, gastrointestinal diseases, vitamin deficiencies etc.… Strikingly, IPF was identified as the second predictor of MDS diagnosis, contributing to 33% of diagnoses (Fig. 2A), followed by ANC (18%). Using a bootstrapping approach, we cross-validated several models and compared their performances, starting from the previously published MDS-CBC score, which includes three parameters. We could have chosen a different approach, starting from scratch and introducing parameters one by one, beginning with the parameter with the highest VIMP after Ne-WX, namely IPF. Our decision was guided by the fact that the MDS-CBC score is already implemented in a wide number of laboratories and that IPF is a parameter only available when a fluorescence platelet count (PLT-F) is triggered by analytical interferences on MPV, profound thrombocytopenia or internal rules of the laboratory (detection of cryoglobulins in case of previously unknown thrombocytosis for example). Comparing performances of the different models starting from three parameters and increasing to six (excluding MPV for being inconstantly available) revealed that the best models were those including IPF, alone or with the platelet count, in addition to the three parameters from the MDS-CBC score. Given the medico-economic issue and the fact that fluorescence platelet counting with the PLT-F channel represents an additional and significant cost for the laboratory, we finally propose a two-step decision tree (Fig. 3D). We suggest slightly modifying the previously published threshold of the MDS-CBC score to 0.23 to trigger a fluorescence platelet counting (if not already triggered by other rules). Systematic fluorescence platelet counting would have identified eleven additional MDS patients with a low MDS-CBC score but probably with a significant cost on unselected cohorts of patients. If the MDS-CBC score is superior to 0.6, in our experience at least, IPF measurement has no additional value for MDS diagnosis. Between 0.23 and 0.6, IPF drastically increases the specificity of MDS-CBC score, limiting the number of slide reviews necessary according to a threshold of 3%. Implementation of MDS-CBC score to trigger slide review in case of cytopenia is a significant progress in the MDS work-up, especially when compared to the cytopenia thresholds from the R-IPSS, which would have missed 22% of MDS and generated unnecessary slides in 47% of non-MDS patients from our cohort.

As mentioned earlier, the main limitation of our study is related to the fact that our cohort was constituted of patients with cytopenia and a genuine suspicion of MDS. This is an advantage when considering the specificity of the score (which would have been higher with unselected controls with for example anemia due to blood loss or iron deficiency) but a disadvantage to evaluate the economic impact of additional PLT-F testing triggered by the decision tree. This point would benefit from being evaluated in non-hospital laboratories with a different case-mix of patients. Another point is that smear review in non-MDS patients may provide crucial information and hints for further work-up (for example pseudothrombocytopenia, giant or abnormal platelets, hairy cells, abnormal red blood cells etc.…). Of course, MDS-CBC score in its initial or extended version is only a help for rationalizing smear review and each clinical laboratory must have its own rules for slide review in agreement with the international or local guidelines and the addition of the extended MDS-CBC score into the routine workflow does not aim to preclude slide review in cases such as neutrophil count < 1,5 × 10^9^/L or thrombocytopenia < 100 × 10^9^/L, for example.

## Conclusions

Data from MDS patients with a low MDS-CBC score emphasizes the fact that platelet dysplasia is probably an early driver event in MDS, the diagnostic performance of which is currently underestimated in routine practice. Deeper exploration of dysplastic thrombopoiesis, by either developing or exploiting platelet-related CPD on routine analyzers could further improve MDS diagnosis on a CBC and slide review efficiency.

## Supplementary Information

**Additional file 1.**

## Data Availability

All data generated or analyzed during this study are included in this published article and its supplementary information files.

## Abbreviations

ANC: Absolute neutrophil count
CART: Classification and regression tree
CBC: Complete blood count
CPD: Cell population data
EDTA: Ethylenediaminetetraacetate
IPF: Immature platelet fraction
MCV: Mean corpuscular volume
MDS: Myelodysplastic syndromes
MPV: Mean platelet volume
R-IPSS: Revised international prognostic scoring system
RBC: Red blood cells
RF: Random forest
ROC: Receiver operating characteristic
VIMP: Variable importance
WHO: World health organization
