# Supplementary material for: Machine learning-based improvement of MDS-CBC score brings platelets into the limelight to optimize smear review in the hematology laboratory
Source: BMC Cancer. 2022 Sep 10;22:972. doi: 10.1186/s12885-022-10059-8 (PMC9464379; doi:10.1186/s12885-022-10059-8)
Supplement: Supplementary file 1 — Additional file 1. [file 12885_2022_10059_MOESM1_ESM.docx]

**Machine learning-based improvement of MDS-CBC score brings platelets into the limelight to optimizes smear review in the hematology laboratory.**

**Jaja Zhu^1^, Pierre Lemaire^2^, Stéphanie Mathis^2^, Emily Ronez^1^, Sylvain Clauser^1^, Katayoun Jondeau^3^, Pierre Fenaux^4^, Lionel Adès^4^ and Valérie Bardet^1^, on behalf of the Groupe Francophone des Myélodysplasies.**

**^1^ Service d’Hématologie-Immunologie-Transfusion, APHP.Paris Saclay, Université Versailles Saint Quentin-Université Paris Saclay, France.**

**^2^ Service d’Hématologie biologique, Hôpitaux Universitaires Saint Louis, Lariboisière, Fernand Widal, Université Paris Diderot, France**

**^3^ Unité fonctionnelle d’Hématologie clinique, Service de Médecine Interne, APHP.Paris Saclay, Université Versailles Saint Quentin, France.**

**Versailles Saint Quentin- Paris-Saclay, France.**

**^4^ Service d’Hématologie Seniors, Hôpitaux Universitaires Saint Louis, Lariboisière, Fernand Widal, Université Paris Diderot, France**

**Correspondence:**

**Pr Valérie Bardet, Service d’Hématologie-Immunologie-Transfusion, Hôpitaux Universitaires Paris Ile De France Ouest, Université Versailles Saint Quentin, France.**

**e-mail :** [**valerie.bardet@aphp.fr**](mailto:Valerie.bardet@aphp.fr)

keywords : myelodysplastic syndromes, smear review, dysplasia score, Ne-WX, MDS-CBC score, macroplatelets, immature platelet fraction

**Supplementary material**

**Figure 1. Violin plots of the basic characteristics of the cohort,** presented by lineage including cell population data (CPD): leukocytes A to C, red blood cells D and E, platelets F to H, MDS-CBC score I and J.

A. White blood cell count (WBC) is significantly lower in MDS patients compared to non-MDS patients (3.5 versus 8.3 x 10^9^/L, p<10^-4^). B. Absolute neutrophil count (ANC) is significantly lower in MDS patients compared to non-MDS patients (1.9 versus 5.5 x 10^9^/L, p<10^-4^). C. Ne-WX is significantly increased in MDS patients compared to non-MDS patients (405 versus 321, p<10^-4^). D. Hemoglobin level is significantly lower in MDS patients compared to non-MDS patients (96 versus 105 g/L, p=0.0007). E. Mean corpuscular volume (MCV) is significantly higher in MDS patients compared to non-MDS patients (94 versus 87 fL, p<10^-4^). F. Platelet count (PLT) is significantly lower in MDS patients compared to non-MDS patients (113 versus 438 x 10^9^/L, p<10^-4^). G. Mean Platelet Volume (MPV) is significantly higher in MDS patients compared to non-MDS patients (11.4 versus 9.8 fL, p<10^-4^). H. Immature platelet fraction (IPF) is significantly higher in MDS patients compared to non-MDS patients (8.3 versus 2.1%, p<10^-4^). I. MDS-CBC score is significantly higher in MDS patients compared to non-MDS patients (0.754 versus 0.016, p<10^-4^). J. Values of the MDS-CBC score depending on the MDS subtype.


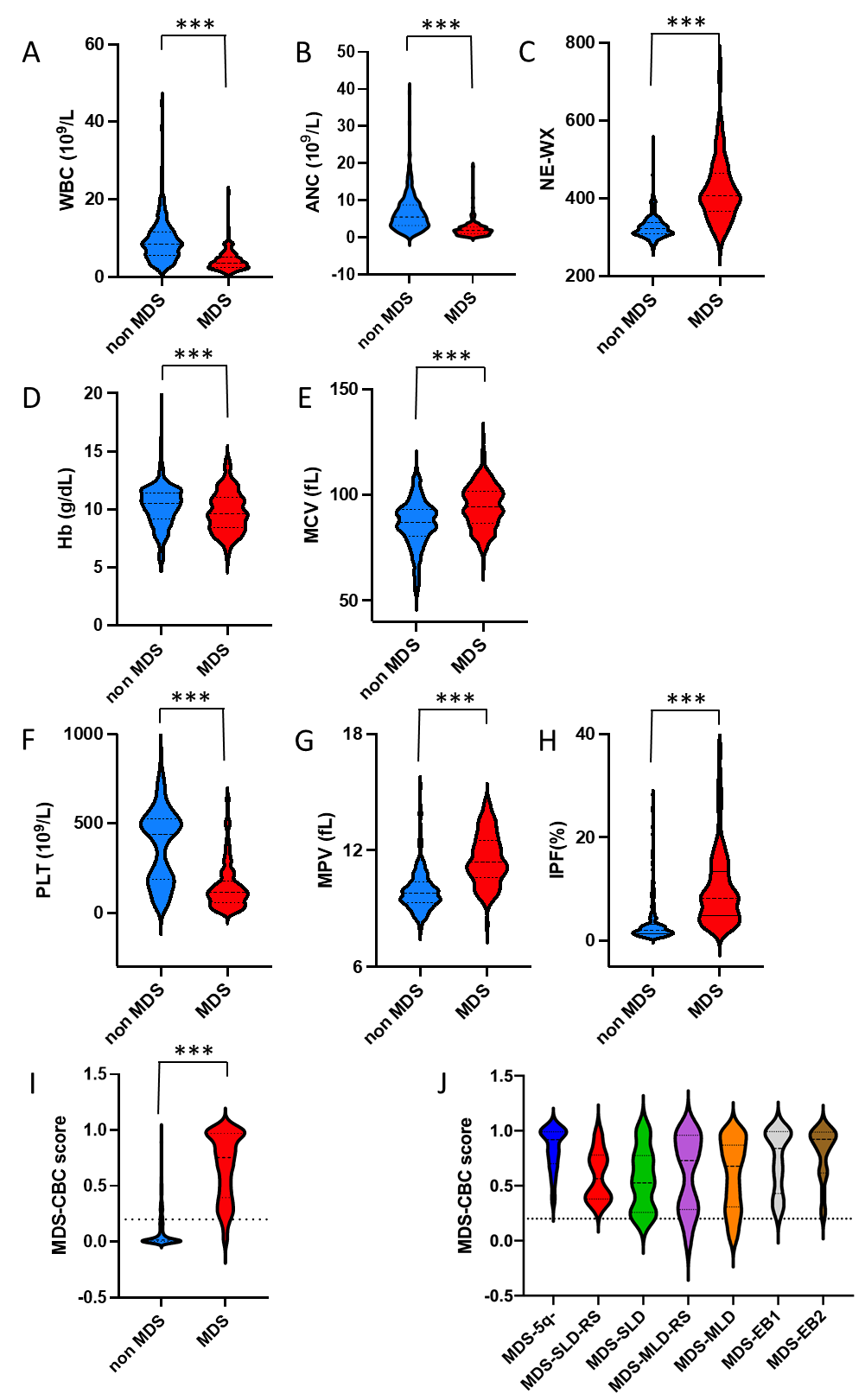


**Supplementary Table 1. Univariate analysis of basic characteristics of the study population**

| Parameter (Median, Range) or n (%) | Non-clonal cytopenias  n=357 | Myelodysplastic syndromes, n=168 | p-value |
| --- | --- | --- | --- |
| Age (years) | 71 [62 - 80] | 78 [71 - 84] | <10-4 |
| Gender (male/female) | 126/231 (35/65) | 90/78 (54/46) | <10-4 |
| Leukocytes (10^9^/L) | 8.3 [5.5-11.4] | 3.5 [2.3-5.0] | <10-4 |
| Absolute Neutrophile Count (ANC, 10^9^/L) | 5.5 [3.2-8.8] | 1.9 [1.0-2.8] | <10-4 |
| Lymphocytes (10^9^/L) | 1.4 [0.9-2.1] | 1.3 [1-1.9] | 0.792 |
| Monocytes (10^9^/L) | 0.6 [0.4-0.8] | 0.6 [0.3-1] | 0.595 |
| Hemoglobin (g/L) | 105 [92-115] | 96 [84-110] | 0.0007 |
| Mean Corpuscular Volume (fL) | 87 [80-93] | 94 [87-102] | <10-4 |
| Platelets (10^9^/L) | 438 [185-526] | 113 [54-177] | <10-4 |
| Mean Platelet Volume (fL), n=478 | 9.8 [9.3-10.4] | 11.4 [10.6-12.5] | <10-4 |
|  |  |  |  |
| NE-WX | 321 [309-338] | 405 [365-464] | <10-4 |
| IPF (%) | 2.1 [1.4-3.2] | 8.3 [4.8-13.3] | <10-4 |
| MDS-CBC score | 0.016 [0.004-0.129] | 0.754 [0.396-0.971] | <10-4 |
|  |  |  |  |
| ANC <1.8 10^9^/L | 36 (10.1) | 77 (45.8) | <10-4 |
| Hb < 120 (F) or 130 (M) g/L | 335 (93.8) | 157 (93.4) | 0.712 |
| Platelets <150 10^9^/L | 28 (18.5) | 112 (66.7) | <10-4 |
| Analyser flag | 27 (7.6) | 62 (39) | <10-4 |
|  |  |  |  |
| Cytogenetics ; n=263 | **n=95** | **n=168** |  |
| Clonal karyotype | 0 (0) | 94 (56) |  |

**Supplementary Table 2. Characteristics of the MDS cohort**

| MDS subtype | All (n=168) | MDS-CBC ≥ 0.2 (n=158) |
| --- | --- | --- |
| MDS-SLD | 12 (7.1) | 12 (100) |
| MDS-SLD-RS | 8 (4.8) | 8 (100) |
| MDS-MLD | 62 (36.9) | 54 (87) |
| MDS-MLD-RS | 16 (9.5) | 14 (87) |
| MDS-del 5q | 16 (9.5) | 16 (100) |
| MDS-EB1 | 31 (18.5) | 31 (100) |
| MDS-EB2 | 23 (13.7) | 23 (100) |
|  |  |  |
| R-IPSS cytogenetic risk group |  |  |
| Very good | 7 (4) |  |
| Good | 111 (66) |  |
| Intermediate | 20 (12) |  |
| Poor | 14 (8) |  |
| Very poor | 16 (10) |  |
|  |  |  |
| R-IPSS |  |  |
| Very low | 46 (27) |  |
| Low | 52 (31) |  |
| Intermediate | 24 (14) |  |
| High | 25 (15) |  |
| Very high | 21 (13) |  |
